# Compression Behavior of EBM Printed Auxetic Chiral Structures

**DOI:** 10.3390/ma15041520

**Published:** 2022-02-17

**Authors:** Kadir Gunaydin, Halit Süleyman Türkmen, Alessandro Airoldi, Marco Grasso, Giuseppe Sala, Antonio Mattia Grande

**Affiliations:** 1 General Electric Aviation, Gebze, Kocaeli 41400, Turkey; 2 Aeronautics and Astronautics Faculty, Istanbul Technical University, Maslak, Istanbul 34467, Turkey; halit@itu.edu.tr; 3 Department of Aerospace Science and Technology, Politecnico di Milano, Via La Masa 34, 20156 Milano, Italy; alessandro.airoldi@polimi.it (A.A.); giuseppe.sala@polimi.it (G.S.); antoniomattia.grande@polimi.it (A.M.G.); 4 Department of Mechanical Engineering, Politecnico di Milano, Via La Masa 34, 20156 Milano, Italy; marcoluigi.grasso@polimi.it

**Keywords:** additive manufacturing, EBM, auxetic, chiral, energy absorption, FEM

## Abstract

In this study, the cyclic compression and crush behavior of chiral auxetic lattice structures produced from titanium alloy (Ti6Al4V) metallic powder using electron beam melting (EBM) additive manufacturing technology is investigated numerically and experimentally. For material characterization and understanding the material behavior of EBM printed parts, tensile and three-point flexural tests were conducted. Log signals produced during the EBM process were investigated to confirm the stability of process and the health of the produced parts. Furthermore, a compressive cyclic load profile was applied to the EBM printed chiral units having two different thicknesses to track their Poisson’s ratios and displacement limits under large displacements in the absence of degradation, permanent deformations and failures. Chiral units were also crushed to investigate the effect of failure and deformation mechanisms on the energy absorption characteristics. Moreover, a surface roughness study was conducted due to high surface roughness of EBM printed parts, and an equation is offered to define load-carrying effective areas to prevent misleading cross-section measurements. In compliance with the equation and tensile test results, a constitutive equation was formed and used after a selection and calibration process to verify the numerical model for optimum topology design and mechanical performance forecasting using a non-linear computational model with failure analysis. As a result, the cyclic compression and crush numerical analyses of EBM printed Ti6Al4V chiral cells were validated with the experimental results. It was shown that the constitutive equation of EBM printed as-built parts was extracted accurately considering the build orientation and surface roughness profile. Besides, the cyclic compressive and crush behavior of chiral units were investigated. The regions of the chiral units prone to prematurely fail under crush loads were determined, and deformation modes were investigated to increase the energy absorption abilities.

## 1. Introduction

Lightweight structures such as sandwich structures play a crucial role in aviation, automotive and military applications, due to the importance of crush and compression strength at impact and blast conditions. Various types of sandwich structures having different types of cores, for instance, lattices, foams and trusses, have been proposed for crashworthy structures. In terms of crashworthiness, the lattice cores have come to the fore, and auxetic solid cellular structures are among the most promising types of lattice structures studied in recent years [1]. Auxetic lattice solid structures reach a negative Poisson’s ratio by showing a significantly different behavior from conventional materials: shrinkage under a compressive load and expansion under a tensile load.

The term auxetic was initially offered by Evans [1] in the year 1991 after Lake [2] introduced the first known synthetic foam structured material with a negative Poisson’s ratio (NPR). NPR materials also can be termed metamaterials, since they are engineered from conventional materials by adopting specific topologies [3,4,5,6]. Indeed, Poisson’s ratios of a chiral auxetic lattice structure is a topological specification that is independent of the structure’s material according to the theoretical approach if the material is isotropic [7]. The special properties of auxetic structures can be obtained through topological configurations. Adjusting different specifications can be done by modification of the unit cell, which is the smallest repetitive domain of the lattice network. At the metamaterial level, mechanical properties such as Young’s Modulus, Poisson’s ratio, sound absorption coefficient and the coefficient of thermal expansion can be adjusted by tailoring the unit cell. In addition, auxetic materials have some special properties, such as high global flexibility without strain localization, improved fracture toughness, transverse shear moduli and most importantly, indentation resistance [8]. Moreover, auxetic lattices can be formed as doubly curved and dome shaped structures. Due to the listed highly remarkable specifications, auxetic structures have been exploited in several applications in aerospace, automotive and defense industries [9,10,11,12]. Different types of auxetic lattice cells can be found in the literature [13,14,15,16].

Among the auxetic lattice structures, chiral lattice structures are one of the most known auxetic lattice structures experiencing a Poisson’s ratio of −1. The structural chiral auxetic honeycomb concept was initially proposed by Prall and Lakes, and the Poisson’s ratio of −1 was proven in a theoretical way based on standard beam theory in their study [7]. Due to its non-centrosymmetric topology, chiral cellular solids exhibit anisotropic behavior, and auxeticity occurs in the edgewise direction (in-plane), so research on it can be divided into two classes, which are edgewise and flat (out-of-plane) studies. Spadoni et al. [17] investigated global linear buckling behavior numerically in the flatwise direction; an FEM model was used in this study with classical theoretical equations for buckling of shells and thin plates while considering the possible formation of localized plasticity. Lorato et al. [18] developed analytical and FEM models to calculate the flatwise Young’s moduli and shear stiffness values of trichiral, tetrachiral and hexachiral lattice configurations, and the models were validated with experimental results. A flatwise dynamic compression study was conducted by Lu et al. [19] to investigate energy absorption behavior, and novel hierarchical chiral lattice elements were presented and adjusted to reach special structures with better energy absorption capabilities.

A number of edgewise studies have been conducted, one of which involved a mathematical model using dynamic shape functions to explain dynamic behavior over a broad frequency range [20]. Additionally, several studies have been conducted on the wave propagation characterization of chiral structures [21,22,23,24]. Chiral structures can maintain their auxeticity under large overall displacement within the elastic deformation limits; however, when they are subjected to large deformations causing plastic deformation, their auxeticity deceives. Zhu et al. [25] used wavy ligaments in their study to increase the auxeticity limit of chiral cells for large elasto-plastic deformation, and as a result, the new structure experienced better performance. Another auxeticity study was conducted by Alderson et al. [3] to elaborate on the elastic properties and auxetic behavior of different chiral cells having 3, 4 or 6 ligaments connected to one node. Additionally, a numerical and experimental study was performed to investigate the elastic and yield onset behavior of achiral and chiral lattice structures, both having six-fold rotational symmetry, in 2D [26]. Furthermore, homogenization studies were conducted for chiral lattices to obtain computationally low cost models [4,27,28]. Despite the peculiar properties of auxetic metamaterials that depend on the topologies adopted, the material of the cellular structures is important in the determination of the mechanical performance for mechanical properties and energy absorption capabilities. Moreover, the production method indirectly plays an important role in cost, production challenges and efficiency. Commercial production of metal-based honeycomb structures includes cutting and bending of aluminum sheet rolls [29]. The advancements in the processes of additive manufacturing (AM) in the past few years have offered the opportunity to generate arbitrary typologies with fewer constraints compared to conventional methods of production. One of the important AM approaches is EBM additive manufacturing, which is using an electron beam to melt and to fuse metallic powders [30]. To sum up, several studies on chiral auxetics have been conducted. However, to the authors’ knowledge, there have been no studies on metal chiral cellular solids and their cyclic and crush performances, along with failure analysis. To fill in that gap and investigate the performance of EBM printed chiral auxetics via failure analysis, this study was performed.

In this research, EBM AM technology was utilized to produce tensile and three-point flexural specimens and lattice units of the chiral auxetic structures to evaluate the impacts of mechanical properties stemming from the production method. All bending and tensile specimens were produced in two different thicknesses of 0.8 and 1.6 mm and orientations of perpendicular (0${}^{\circ }$) and parallel (90${}^{\circ }$) to the build plate of the EBM machine. Chiral units were manufactured with their flat-sided surfaces parallel to the plane of powder deposition to achieve higher mechanical efficiency and avoid the use of internal support structures. Two different thicknesses were adopted for chiral structures to characterize the effects of thickness of chiral units on auxeticity, stiffness and energy absorption. The compressive load profile was applied to chiral structures to monitor their Poisson’s ratio variations under different deformation limits, and a crushing load was applied to investigate the effects of failure and deformation modes of chiral units’ crush absorption characteristics. A material characterization study was conducted with tensile and bending tests, including specimens with different thicknesses and build orientations. In addition to a material characterization study, surface roughness profiles of the specimens produced in vertical and horizontal orientations with different thicknesses were studied, and an equation is offered here to find the load-carrying effective areas of such specimens. In compliance with the equation and tensile test results, a constitutive equation was formed and used after selection and calibration process to verify the numerical model for optimum topology design and mechanical performance forecasting using a non-linear computational model with failure analysis. The numerical analyses that were used for the simulation of cyclic compression and crush tests were validated with the experimental results. The results show that the equation of EBM printed as-built parts obtained from tensile test results and surface roughness assessments captures the experimental results accurately. In addition, the regions of the chiral units prone to prematurely failing under crush loads were determined, and deformation modes were investigated to increase the energy absorption abilities. The effects of ligament thickness on the Poisson’s ratio and crush performance were also investigated.

## 2. Production and Characterization of Additively Manufactured Chiral Elements

### 2.1. Chiral Cellular Structures

**Figure 1:**
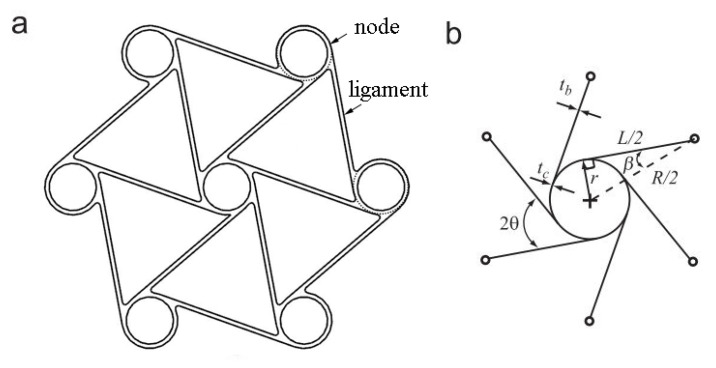
Hexagonal chiral element: (**a**) representative unit and (**b**) geometrical parameters.

A chiral auxetic solid network can be described by its interconnected nodes and ligaments structures; each end of a ligament is tangentially linked to a node, as shown in Figure 1a. The number of ligaments linked to one node identifies the chiral lattice structures type as trichiral, tetrachiral or hexachiral structures for 3, 4 or 6 tangential ligaments connected to one node, respectively. The anti prefix for chiral auxetic structures is meant to define the spatial relationships among chiral network nodes and ligaments. Chiral lattice auxetic structures with cylindrical nodes facing the direction opposite the ligament tips are called chiral network systems; when cylinders run in the same direction as the ligament, they are called antichiral systems. Nevertheless, not all chiral network systems exhibit negative Poisson’s ratios; 4 and 6 ligament linked systems and short ligament-limited anti-trichiral network systems just have auxeticity [31]. The chiral hexagonal auxetic lattice structure was selected for this study, which exhibits full-wave shaped ligament deformation. The chiral hexagonal unit and topology parameters are shown in Figure 1. The structure consists of equal-sized cylinders, or nodes, linked by the ligaments of the same length *L*. The extrusion length of the chiral unit is indicated by *e*. The nodes’ outer radii are denoted by *r*, and the negative Poisson’s ratio is generated by the bending of the ligaments around the nodes. Chiral system deformation begins with node rotation, and ligament bending as a result of node rotation. During the deformation of the chiral unit, two different ligament deformations can be seen according to the type of chiral network system. The full wave-shaped ligament deformations can occur in the chiral systems and half-wave shaped ligament deformations in the anti chiral network systems. The full-wave shaped ligaments absorb more energy in crashworthiness and compression applications [3].

The angle of θ and β are the topology parameters, and their relationships between other parameters are given in Equation (1).

(1) $$\sin\;\beta =\frac{2r}{R},\;\cos\;\beta =\frac{L}{R},\;\tan\;\beta =\frac{2r}{L},\;\sin\;\theta =\frac{R/2}{R}=0.5;\;\theta =30$$

Equation (1) defines the topology layout and the ligament orientations with regard to the imaginary line through each node’s center [7,17]. Among the parameters, cosβ has a special place by dominating the chiral geometry significantly. According to the study of Spadoni et al. [32], *L/R* mainly affects the mechanical performance of the chiral structure. In the study of Prall and Lakes, −1 Poisson’s ratio of chiral structure was shown theoretically with a rigid node assumption, and the elastic modulus of a chiral structure is given in Equation (2) [7]. *E_s_* is the Young’s modulus of lattice cell constituent material. To obtain stiffer chiral structures, the thickness of the chiral units can be increased, and the ligament length and radius of nodes can be decreased. Two different chiral units with different thicknesses of 0.8 and 1.6 mm, called C1 and C2 hereafter, respectively, were considered in this study to investigate the effect of thickness. Design parameters of the chiral units are exhibited in Table 1.

(2) $$E=E_{s}\sqrt{3}\;\frac{t^{3}}{L\;r^{2}}$$

Figure 1b shows that there are two parameters for thicknesses: one of them is for node thickness, *t_c_*, and another is for ligament thickness, *t_b_*. Both *t_c_* and *t_b_* were set as equal.

### 2.2. Materials and Processing

**Figure 2:**
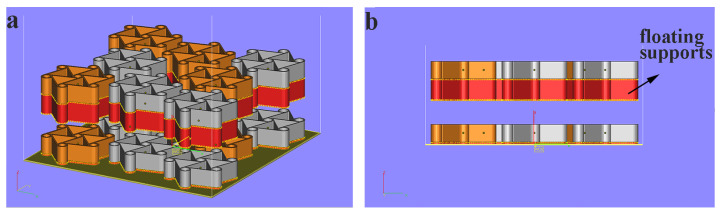
Floating parts production in EBM: (**a**) iso view and (**b**) front view.

**Figure 3:**
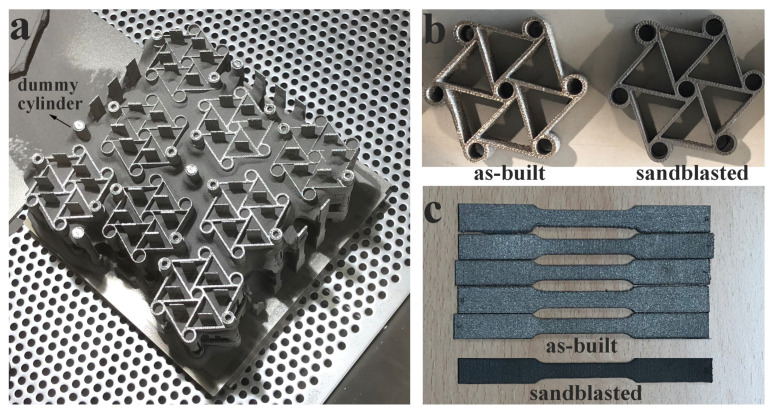
EBM printed parts: (**a**) printed parts in powder cake, (**b**) as-built and sandblasted chiral parts and (**c**) as-built and sandblasted tensile specimens.

In this study, an ARCAM A2 system was used for the additive production of tensile, bending and chiral unit specimens. Sixteen chiral unit samples were produced in the same build by using gas atomized Ti6Al4V ELI (extra low interstitial) powder with an average size of 45–106 μm. Table 2 shows the powder’s chemical composition. The mechanical specifications indicated by the machine vendor for this material are listed as Young’s modulus, yield stress, ultimate tensile strength and elongation equal to 120 GPa, 930 MPa, 970 MPa and 16%, respectively [33]. The samples were produced on two different levels along the z-axis, as shown in Figure 2. By exploiting the pre-sintered nature of the powder, caused by the high-temperature pre-heating phase, “floating supports” were used for samples in the upper z level, as shown in Figure 2b. Dummy cylinders were included in the build to enhance the homogeneity of the thermal load within the entire build volume, as exhibited in Figure 3a. Four groups of tensile and bending specimens, including five specimens in each group, were produced. Groups can be defined as 0.8 mm thick vertical and horizontal, and 1.6 mm thick vertical and horizontal builds. The samples were produced with a layer thickness of 50 μm while applying default process parameters provided by the machine vendor for this powder.

The surface finish is an important factor in the analysis of the mechanical performances of EBM printed parts, as EBM yields rougher surfaces than other AM processes. Polished specimens exhibit better strain at failure values for as-built parts thanks to the mitigation of surface irregularities that may cause crack initiations, and therefore stress concentration and premature failure [34]. Poor surface roughness and the presence of powder particles sintered on the surface may affect the stress calculation because of undesired variability and biases in cross-sectional area estimation. In this study, all samples were sandblasted with a fixed sandblasting cycle duration of 5 s for each surface. Surface roughness measurements were performed before and after the sandblasting operation. Figure 3 shows the samples in the pre-sintered bulk of powder at the end of the EBM process (Figure 3a); the as-built and sandblasted chiral unit components (Figure 3b); and the as-built and sandblasted tensile specimens that were already included in the same build (Figure 3c). In addition, a Hitachi Tabletop Microscope TM3000 was used to measure the effective cross-sectional areas after tensile specimens were cut from the gauge region and polished using a mechanical polisher with 1200 grit sandpaper.

For the characterization analysis presented in this study, the avoidance of nonhomogeneous process conditions in different locations of the build waws of great importance. In particular, before testing the chiral sample performances, we carried out an analysis to verify that process conditions remained stable during the production of samples on the two different levels in z direction. The verification was based on analyzing the so-called “log signals”—i.e., signals measured during the process—and on a layer-by-layer basis by means of sensors embedded in the EBM system. Various authors [35,36] discussed the suitability of these signals for determining the stability of the process and predicting variations in the final quality and performances of the products. These signals enclose information about the vacuum conditions, the regularity of the powder deposition, the energy input to the material, the process parameters adapted by embedded optimization algorithms, etc. A subset of most critical signals was identified, and statistical analysis was performed to compare signals acquired during the production of chiral samples placed at two different z levels.

One first family of log-signals included (i) the pulse length of powder sensors, which indicates the amount of powder spread in each layer; (ii) the grid cup voltage, indicating the voltage used to control the electron emission; (iii) the filament voltage, i.e., the cathode voltage used to generate the beam; (iv) the beam current, indicating the energy input to the material; (v) the beam focus, i.e., the current applied to focus and defocus the beam; (vi) the vacuum level in the chamber; and (vii) the total time needed to process each layer. A shift in one (or more) signal belonging to this family would have indicated a variation in the process conditions that could have had detrimental effects on the homogeneity of the microstructural and mechanical properties of parts built at different levels.

The second family of signals consisted of the number of so-called “scan lengths” in each layer. The EBM controller adapts the beam current and the scan speed along each track as a function of the length of the track. The information about the number of scan lengths in different length ranges is made available by the controller for each layer. A shift in the number of scan lengths is an indication of a local variation of the actual energy density, which may introduce local defects and generate non-uniform microstructural properties.

**Figure 4:**
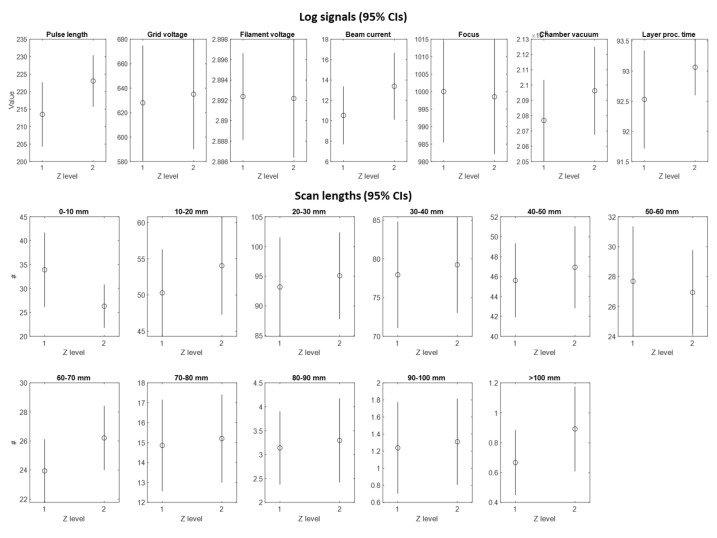
The 95% CIs for the mean log-signals at two different levels along z direction where the chiral samples were produced, where level 1 indicates the bottom level and level 2 indicates the top level.

The two levels in the z plane are denoted as level 1 (bottom) and level 2 (top). Figure 4 shows the 95% confidence intervals (CIs) of the mean values in these two levels for each analyzed signal. The analysis confirmed that none of the considered signals exhibited a statistically significant variation in the mean and standard deviation with a family-wise confidence level of 95%. This allowed us to consider the chiral samples produced at different levels as replicates and to neglect any possible undesired location effect.

### 2.3. Procedures for Mechanical Characterization and Testing

EBM printed parts show anisotropic behavior due to the layerwise production method. Accordingly, the mechanical properties of the EBM printed chiral units can be properly analyzed only if a complete characterization of EBM printed material is carried out. Tensile tests were performed in accordance with ASTM E8M for characterizing EBM printed Ti6Al4V parts and elucidating the mechanical properties. Moreover, three-point bending tests were performed, by adopting a testing jig with a span length of 15 mm, and a pin radius of 1.5 mm was used. The test rate for bending was defined as 1.47 mm/min according to having the same strain rate as the tensile test. The test specimens used in the bending tests were 32×6 mm with 0.8 and 1.6 mm thicknesses. Both tensile and bending test specimens were produced in two different directions, 0${}^{\circ }$ and 90${}^{\circ }$.

**Figure 5:**
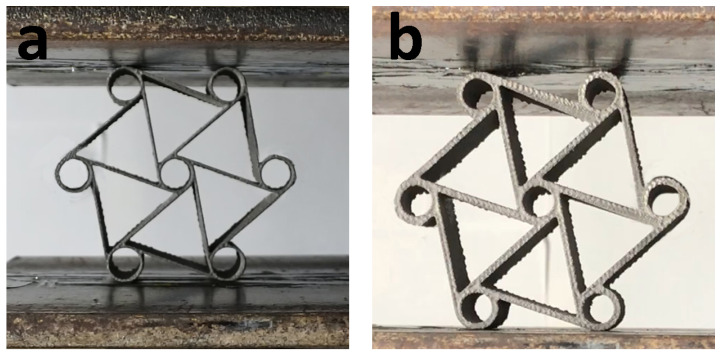
Crush test setup and specimens of chiral units with (**a**) 0.8 mm and (**b**) 1.6 mm ligament thickness.

**Figure 6:**
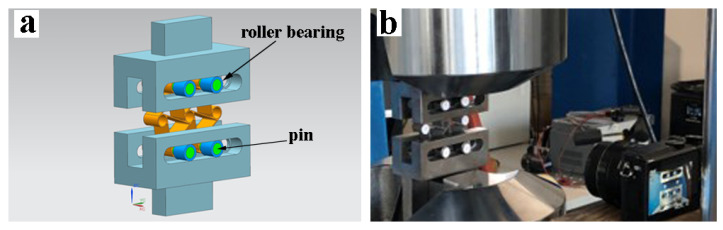
Test setup for cyclic loading showing (**a**) the design and illustration of text fixture and test layout (**b**) produced text fixture and experimental layout.

In all tests, MTS 810 Material Test System (MTS Systems Corporation, Eden Prairie, MN, USA) was used with MTS 647 Hydraulic Wedge Grip (MTS Systems Corporation, Eden Prairie, MN, USA), and MTS 634.31F-24 (MTS Systems Corporation, Eden Prairie, MN, USA) extensometer was utilized in the tensile tests. As for the crush tests, two rigid plates were used to compress chiral units and lubricant applied to the surface of rigid plates to enable the free movement of the chiral elements, as seen in Figure 5. In addition, for the chiral lattice cyclic compression tests, an additional specific fixture was designed to accomplish the tests, which consisted of two wide steel forks, with horizontal grooves. Pins were inserted in the four lower and upper nodes of the chiral element, and roller bearings were attached to the pins to allow them to slide in the grooves. The design and illustration of fixture and the test layout are shown in Figure 6a. Therefore, the two upper and lower nodes of chiral elements were allowed to move laterally and to rotate while the unit was compressed, as seen in Figure 6a. The test velocity was defined as 1 mm/min. Transverse strains were measured to calculate Poisson’s ratio. For measurement of transverse deformation, all tests were recorded with a high definition camera as seen in Figure 6b, and speckled papers were placed on the nodes to track their movements in the cyclic tests.

**Figure 7:**
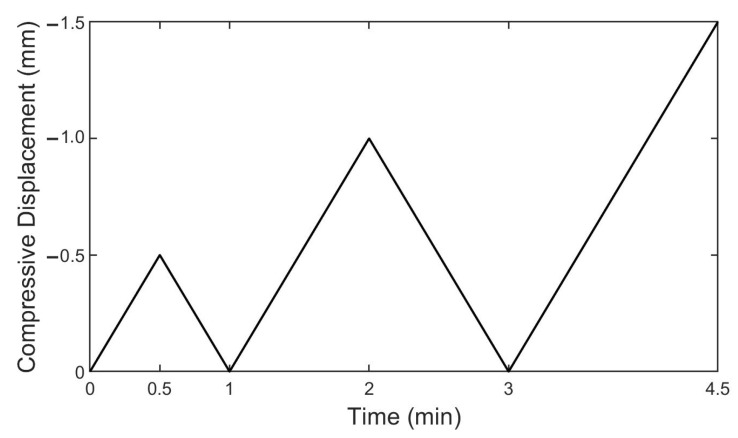
Compressive load profile for chiral lattices.

The chiral elements produced with different thicknesses of 0.8 and 1.6 mm were tested with a compressive load profile to investigate their elastic limits by applying large displacements without experiencing permanent deformations, degradation or failures. The compressive load profile is depicted in Figure 7. The test first started with compression to a limit of 0.5 mm displacement, and thereafter a displacement controlled tensile phase was commenced and continued in which the load reached zero, and then compression started again and proceeded until the defined displacement limit.

## 3. Experimental Results

### 3.1. Results of Material Characterization Tests

All tests were performed after applying a sandblasting process to remove loose particles and decrease the surface roughness. Arithmetical mean deviation (R_a_) of the assessed profiles were found 20.325 μm with a decrease of 5% after sandblasting process. After that, regarding the determination of the effective area, cross-sections from the middle of the gauge section of tensile specimens were extracted and investigated under a microscope.

**Figure 8:**
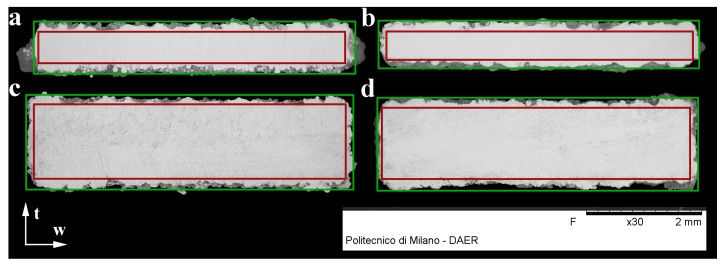
Cross-sections for (**a**) 90${}^{\circ }$, 0.8 mm thickness; (**b**) 0${}^{\circ }$, 0.8 mm thickness; (**c**) 90${}^{\circ }$, 1.6 mm thickness; and (**d**) 0${}^{\circ }$, 1.6 mm thickness specimens.

The green and red rectangular areas show the measured area and effective area, respectively, as seen in Figure 8. The area between red and green area can be considered as mechanically inefficient area, owing to the irregular distribution of this material along the specimen axis. We found that the inefficient area in the thickness (*t*) direction does not vary according to the thickness of produced parts. For as-built parts, the inefficient area thickness was 0.175 mm, and it was 0.165 mm for sandblasted parts. Furthermore, the inefficient area thickness in the width direction shows a similar pattern, and it was 0.130 or 0.120 mm for as-built and sandblasted parts, respectively. As a result, the effective area (A_e_) can be calculated according to Equation (3) for sandblasted parts.

(3) $$A_{e}=\left(t-0.165\right)\times \left(w-0.120\right)$$

Ti6Al4V tensile test specimens were produced according to ASTM E8M in two different directions of horizontal and vertical. Five specimens were produced and tested for each direction and thicknesses. Due to the high surface roughness of EBM printed parts, tensile parts were sandblasted in the same manner before the testings to reduce the surface roughness. In addition, Poisson’s ratio of horizontal test specimens with different thicknesses of 0.8 and 1.6 mm were found 0.3 and for verticals were 0.31.

**Figure 9:**
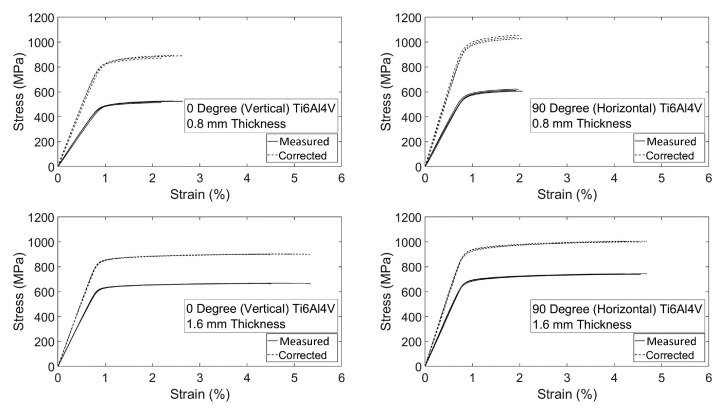
As-built and corrected tensile results for 90${}^{\circ }$, 0.8 mm thickness; 0${}^{\circ }$, 0.8 mm thickness; 90${}^{\circ }$, 1.6 mm thickness; and 0${}^{\circ }$, 1.6 mm thickness specimens.

The tensile test was elaborated to obtain the stress vs. strain curves, considering both the measured and the corrected areas. Results are presented in Figure 9 for 0.8 mm thick and 1.6 mm thick specimens with the vertical and horizontal direction of production. Corrected results indicate the stress values that are calculated by the corrected area using Equation (3) instead of the measured area with a caliper.

**Figure 10:**
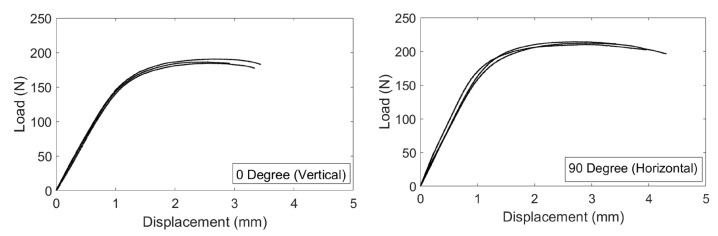
Load-deflection curves for 0${}^{\circ }$ and 90${}^{\circ }$ build orientation specimens in the bending test.

According to the analyses of the corrected tensile results, as shown in Table 3, Young’s moduli do not show considerable variations with building direction and specimen thickness. The values obtained were 110 ± 8 GPa for 1.6 mm thickness and 110 ± 10 GPa for 0.8 mm thickness specimens. The yield stress and ultimate tensile strength values were 847 ± 5 MPa and 901 ± 9 MPa, respectively, for 0${}^{\circ }$ orientation production, and 989 ± 12 MPa and 1040 ± 12 MPa, respectively, for 90${}^{\circ }$ orientation builds for the 0.8 mm thickness specimens. For the 1.6 mm thick specimens, the yield stress and ultimate strength values can be listed as 855 ± 3 MPa and 903 ± 1 MPa for 0${}^{\circ }$ orientation production, and 934 ± 11 MPa and 1005 ± 3 MPa for 90${}^{\circ }$ production. The results show that the direction of production and the thickness of the sample is not a dominant factor for Young’s modulus, but better yields stress and ultimate tensile strength values were experienced in the 90${}^{\circ }$ building orientations. Additionally, low strain at failure values were obtained for 0.8 mm thickness specimens: 0${}^{\circ }$ built specimens exhibit 2.42 ± 0.21% and 90${}^{\circ }$ built shows 1.99 ± 0.07%. The strain at failure values for 1.6 mm thick specimens were better compared to the ones of 0.8 mm thick specimens, with values of 4.93 ± 0.41% and 4.50 ± 0.19% for 0${}^{\circ }$ and 90${}^{\circ }$ building orientations, respectively. The aforementioned company specified mechanical properties superior than these experimental test results. This circumstance can be clarified by the lack of fusion, oxidation, high surface roughness, leftover built support parts and surface deformation stemming from the built support removal process, which is caused by the EBM production method characteristics and material inhomogeneity. However, mechanical properties can be improved by applying post-process such as hot isostatic pressure, vacuum stress relief or ageing. The reason for the difference in strain at failure values for 0.8 and 1.6 mm thicknesses can be explained by the effect of local deformation due to high surface roughness that can promote a premature localization of plastic strain in the smallest sections. The effective load supporting area is greater for 1.6 mm thickness specimens which can endure more under the effect of high local deformations. Finally, the improved yield and ultimate strength of specimen with 90° building orientation were justified by the application of the load in the same direction of the layers melted in the steps of the production process, without involving the stress transmission through adjacent layers. As a result of the tensile study outputs, it is found that EBM printed Ti4Al6V parts by using the building theme proposed by the machine vendor, show isotropic elastic and plastic anisotropic behavior. However, due to the variations in the strain at failure, 90${}^{\circ }$ production demonstrates consistent mechanical performance in comparison to the 0${}^{\circ }$ production orientation. Using Equation (3), three-point flexural test results were re-calculated and given in Figure 10.

### 3.2. Dimensional Conformance

Net shaping is an important phenomenon for production systems, and thus, the dimensions of both C1 and C2 were measured to check dimensional accuracy, and results are exhibited in Table 4. Considering sandblasted part dimensions, the height and nodes outer radius of both C1 and C2 were within the tolerance of 1%; however, extrusion length of chiral units was only within 3% tolerance after sandblasting. The thickness of the ligaments for C1 was 0.97 with a difference of 21%, whereas it was 1.71 for C2 with a difference of 12%. Moreover, the inner radius of nodes for C1 and C2 are 6% lesser than design dimensions. As to as-built parts dimensions, their dimensional difference from the sandblasted parts was nearly 10 mm except the inner diameter of nodes. Sandblasting could not be applied perpendicularly to the node surfaces due to narrow space. According to measured dimensions and Equation (2), C1 and C2 were redesigned and used in FEM analysis with the new dimensions.

### 3.3. Results of Compression and Crushing Tests on Chiral Units

**Figure 11:**
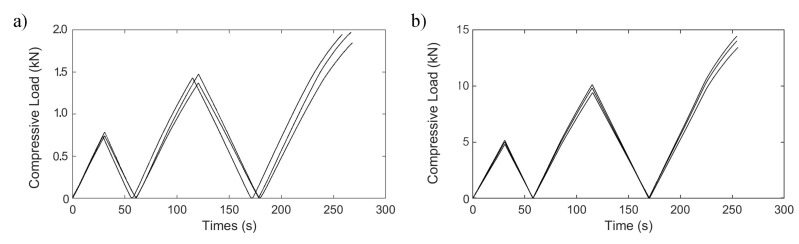
Compressive load profiles: experimental and FEM results for chiral elements of (**a**) 0.8 mm thickness, (**b**) 1.6 mm thickness.

As a result of cyclic compressive test, Figure 11 was obtained. In the first two cycles which had 0.5 and 1 mm displacement limits, permanent contractions were 0.15% and 0.3%, respectively, in the height of the chiral unit was observed for C1. Poisson’s ratio was measured as −0.64 in the first cycle, −0.40 in the second cycle, and −0.32 in the third cycle. The decreasing change in Poisson’s ratio was caused by the nonlinear deformation of the chiral units and a residual displacement at unloading, which can be attributed to plastic deformations that occurred in the regions in which the maximum stress levels emerged. However, no lateral deformation was observed, and consequently, neither a negative nor positive Poisson’s ratio was not found in C2.

**Figure 12:**
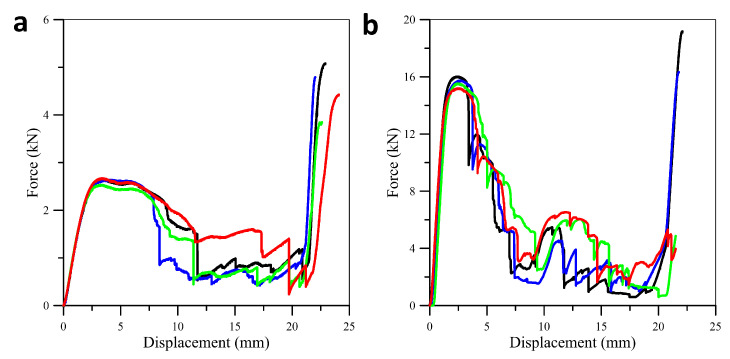
Compressive load and deflection graphs of chiral units with a ligament thickness of (**a**) 0.8 mm, (**b**) 1.6 mm.

The permanent contractions of 0.1% in the first cycle and 0.17% in the second cycle were observed in the height of the chiral unit of C2. The ligaments of the structure were too rigid and the wrapping mechanism of the chiral unit was not achieved. The calculated stiffness value for C2 is 11 KN/mm, and it is 1.45 kN/mm for C1, which validates Equation (2). As a result of the compressive load profile, the chiral unit has a yield stress limit under the compressive loads greater than 1790 N in the last cycle of load profile with a displacement of 1.312 mm for C1, and 10,940 N in the last cycle of the load profile with a displacement of 1.051 mm for C2. In addition, for crush performance of C1 and C2, Figure 12 shows four C1 and C2 specimens crush test results of chiral units as load-deflection curves. To evaluate the crushability of structures, some indexes must be known. During the crushing of cellular structures, three different distinct phases appear and they can be distinguished over the load-deflection curve of cellular structure. These phases are called elastic, plateau and densification phases. In the elastic phase of the cellular structures, mostly linear deformation behavior is exhibited in the load-densification curve and the deformation is elastic. The following phase is the plateau phase and in this phase, mostly plastic deformation occurs. The energy is substantially absorbed in the plateau phase by plastic deformation and fracture. Thus, the elongated plateau phase addresses the higher amount of energy absorption. The last phase is the densification phase in which cellular structure is mostly crushed and starts to act as bulk material with an instant increase in the load-deflection curve. The onset of densification point on the load-deflection curve is a critical point that needs to be determined by numerical methods in order to obtain energy-absorption parameters consistently. The absorbed energy (EA) under crush load is specified as the area under the load-deflection plot and can be calculated via (4).

(4) $$\mathrm{EA}=\int_{0}^{d}Fd\delta$$

In Equation (4), *F* is the reaction force of the cellular structure that generate against crush load and *d* is the deflection of the cellular structures up to the onset of densification point. Regarding the calculation of the onset of densification point over the load–displacement curve, the energy efficiency parameter is required and can be calculated via Equation (5).

(5) $$\eta \left(\delta \right)=\frac{1}{F(\delta )}\int_{0}^{d}F\left(\delta \right)d\delta$$

By means of the energy efficiency parameter, a representative onset deflection of densification can be defined numerically and consistently by obtaining the energy efficiency–deflection plot and selecting the highest point, as expressed in Equation (6) [37]:

(6) $$\frac{d\eta (\delta )}{d\delta }{|}_{\delta =d}=0$$

The main requirements for crush structures are low weight and high energy absorption. Thus, a specific energy absorption (SEA) parameter is used to classify the energy absorption per mass and is displayed in Equation (7).

(7) $$\mathrm{SEA}=\frac{\mathrm{EA}}{m}$$

In addition, mean crushing force (MCF) is another crush efficiency parameter to be taken into account to evaluate crush ability of a structure.

(8) $$\mathrm{MCF}=\frac{1}{d}\int_{0}^{d}Fd\delta$$

Mean crushing force is divided by peak crushing force (PCF), the highest force in the load-deflection curve, to obtain crushing force efficiency (CFE), which should be close to 1 [38], as defined in Equation (9).

(9) $$\mathrm{CFE}=\frac{\mathrm{MCF}}{\mathrm{PCF}}$$

In the first phases in which elastic deformation and bending of the ligaments occurred in between 2.50 and 2.70 kN for C1, it was followed by the collapse of the chiral units, which experienced an acceptable plateau phase. In almost half of the plateau phase, the peak force was kept, and a slight reduction was observed that directly affected the CFE value. The densification phase in which the structure acts as a bulk material started in between 20 and 22.5 mm strokes. As for C2 specimens, they experienced better consistency in their response to crush load; however, the collapse of the chiral units started prematurely, which caused a low CFE value. The peak value of C2 specimens reached almost 6 to 7 times the base, which was already expected from Equation (2). Moreover, the plateau phase of the C2 chiral units was not in an acceptable range. The crushing indexes of both structures can be seen in Table 5. According to the table, it can be said that even if the SEA value of C2 is better than that of C1, the CFE of C2 is not in a feasible range. However, both structures experience similar densification displacements.

## 4. Numerical Analysis

### 4.1. Numerical Models of Chiral Units

**Figure 13:**
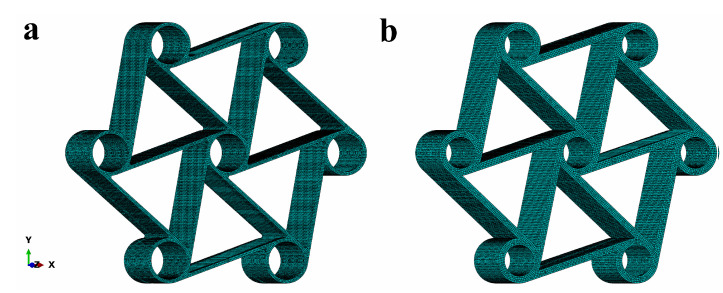
Finite element meshes of (**a**) C1 and (**b**) C2.

For numerical analysis of the three steps, cyclic compression of C1 and C2, implicit finite element models were developed by using the Simulia/Abaqus Standard finite element code. In the analyses, quadratic brick elements (C3D20R) having a reduced integration scheme were used. During the meshing process, a relatively irregular mesh was obtained due to the complex shape of the chiral structures. The maximum deviation factor was kept under the value of 0.1 and the maximum aspect ratio was defined as 3. The selection of appropriate element types aimed to provide better accuracy in the validation by maintaining lower computational costs. Thus, quadratic elements were selected and convergence studies have been conducted. As a result, approximately 193,154 continuum elements for C1 and 214,360 for C2 were created, having typical sizes of 0.3 and 0.4 mm for C1 and C2, respectively. The finite element models of the two chiral C1 and C2 elements are presented in Figure 13.

The elastic and plastic deformation under cyclic loading of C1 and C2 structures was assessed using non-linear analysis. Hence, non-linear material was used by adopting a conventional elastic-plastic material model, with plasticity associated with Von Mises Criterion. The material model was calibrated by using the results obtained in the tensile and bending tests. Non-linear analyses of cyclic loading were conducted by using the Simulia/Abaqus Standard solver, by imposing displacement and load boundary conditions [39]. Self contact interaction with the penalty algorithm was used to model the possible mutual contacts between elements and nodes. To simulate the free rotation of the chiral nodes constrained through the pins, a distributing coupling was used.

**Figure 14:**
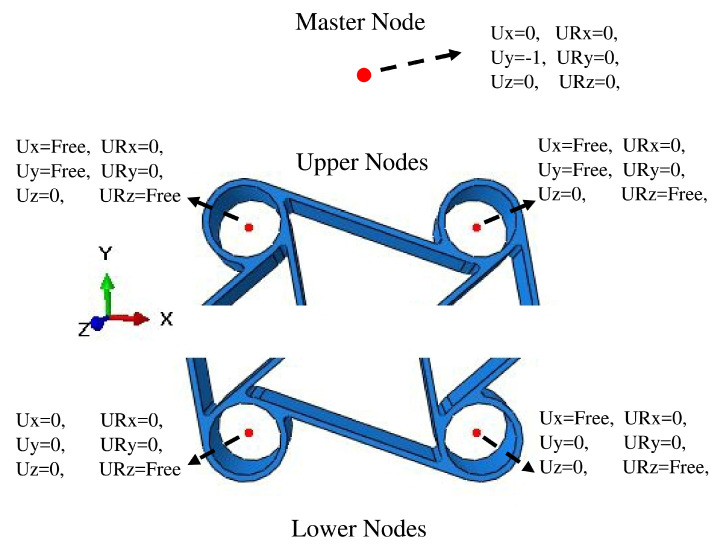
Boundary conditions of chiral units for cyclic displacement.

Distributing couplings were used to constrain the motion of coupling nodes over the rotation and translation of the reference node, and this link enabled the control of transmitted loads using weight factors at the coupling nodes [40]. For the distributing couplings, reference nodes were created in the middle of the cylindrical chiral nodes, and the nodal degrees of freedom of the elements belonging to the inner surface of the chiral nodes were linked to the motion of such reference nodes. In particular, the nodes of the inner surfaces were forced to rotate according to the rotation of the reference node, but the surface could deform radially. These reference nodes are shown in Figure 14. The boundary conditions applied to the upper nodes made possible rotation about the pin axes (z-axis) so that the reference node and the linked surface of the chiral node were free to rotate; transverse motion was available in x-direction and the vertical motion in the y-direction. For the lower ones, rotation about pin axes was free, but vertical translation was set to zero and the horizontal translation of one of the nodes was fixed, to make a statically determinate, constrained system. The vertical compression was applied by connecting the vertical translation of the two upper nodes to the one of an additional master reference node, which was moved downward, as indicated in Figure 14. Moreover, a subroutine was implemented to define the load and displacement control at the same time for the compressive load profile. Whenever compression is applied to the chiral elements, some elements undergo plastic deformation even under small displacements. This situation caused a tensile load while reference nodes were trying to move the initial coordinates.

Regarding the FEM crush analyses of cellular chiral solids, Abaqus/Explicit FEM code was utilized. For the element in the analyses, an eight-node linear hex (C3D8I) element was used, which is an improved element having incompatible modes for the purpose of overcoming pure bending simulations. Quadratic brick elements (C3D20R) that are used in implicit cyclic analysis cannot be used in the explicit analysis due to the order of the elements. Thus, a linear element was deployed in the explicit analysis. The same meshes developed for cyclic compression analyses were used. In terms of material model, besides the linear elastic model, the classical plasticity model for metal materials was utilized with progressive damage evolution law and ductile damage initiation criterion for capturing both plastic behavior and failure of the chiral units [40,41]. The corrected true stress–strain test results were utilized to extract the required damage initiation parameters. The fracture strain and stress triaxiality were defined as 0.021 and 0.33 in accordance with the tensile test results. Besides, the strain rate is another input that had to be specified for damage initiation; but in the condition of material has strain-independent behavior or in case that the strain rate is the same as the material characterization strain rate, the strain rate parameter is specified as zero. Furthermore, the strain at fracture and strain triaxiality had to be specified, and the strain at fracture is defined as the total strain value on the load–displacement curve that corresponds with the ultimate strength achieved. The rate of the hydrostatic pressure stress to the equivalent stress provided the needed strain triaxiality parameter. The strain triaxiality was set as 0.33 due to the acceptance of no shear stress and transverse strain occurs in the uniaxial tensile test until the ultimate strength is reached. Moreover, the damage evolution parameter was also required, and it was specified by the total energy in the tensile test that deforms the specimen until the region that necking occurs. In addition, the fracture energy had to be specified for each FEM element in accordance with its average length between each node, which is called characteristic element length. Thus, the elements used in the discretization of the models were to be kept of equal size to provide the same characteristic element length. At last, displacement at failure value had to be specified, and it is the rate of fracture energy to ultimate tensile strength, which we defined as 0.001 [40]. For the self-interaction or interaction of the parts with different parts in the simulation, general contact including the penalty algorithm was utilized. The same boundary conditions were applied to represent the constraint between the unit and the testing fixture.

### 4.2. Numerical Results and Correlation

According to measured dimensions and Equation (2), C1 and C2 redesigned, and used in FEM analysis with the new dimensions.

**Figure 15:**
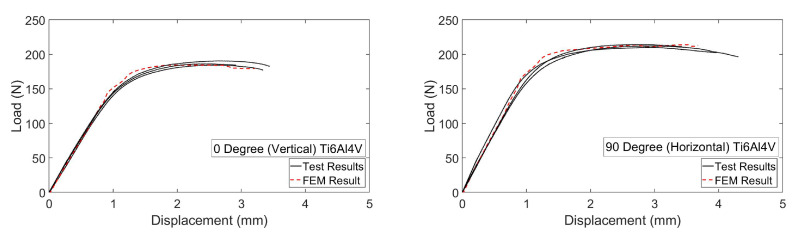
Load-deflection curves for 0${}^{\circ }$ and 90${}^{\circ }$ build orientation specimens in the bending test.

**Figure 16:**
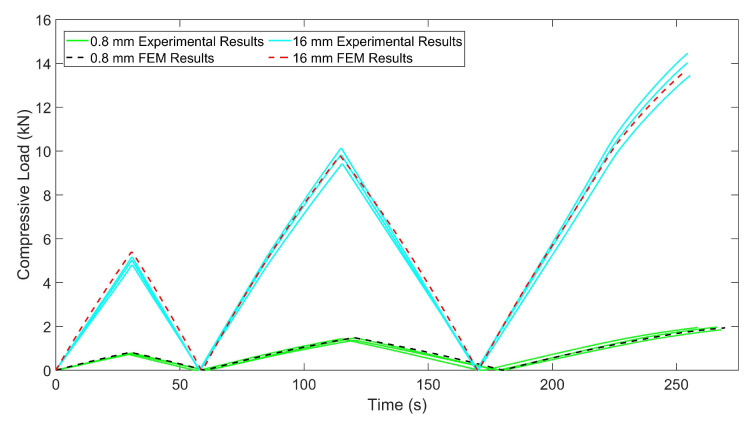
Compressive load profile experimental and numerical results for chiral units with 0.8 mm and 1.6 mm thicknesses.

Consequently, parameters for the constitutive equation were acquired, adjusted and imported into FEM codes as a consequence of experimental studies to constitute the material’s behavior. In the FEM analysis, isotropic elasticity and plasticity were used to validate flexural test results and to measure the deformation limit of chiral units without experiencing permanent deformations, degradation or failures. As seen in Figure 15, close correlations were monitored between flexural test and numerical analysis results. Furthermore, as a result of the chiral compressive load tests and FEM analysis, Figure 16 was obtained.

**Figure 17:**
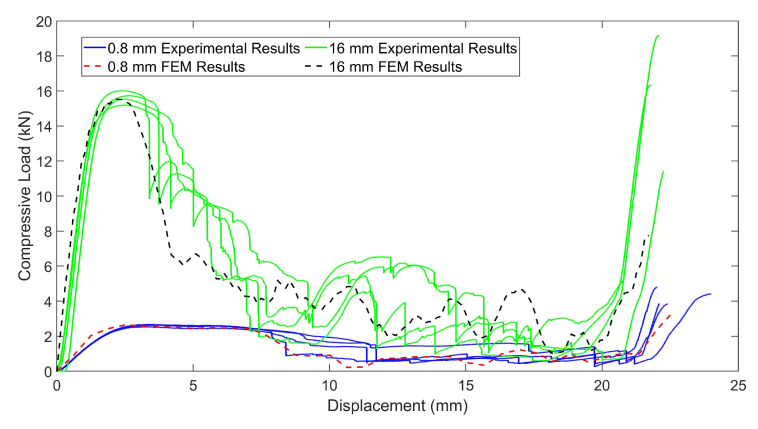
Experimental and numerical results for the crushing of chiral units with 0.8 mm and 1.6 mm thicknesses.

In Figure 17, the crush results of C1 and C2 for experimental tests and FEM analysis are presented. The correlation between numerical and experimental results are in the acceptable ranges, so the numerical models can be used for simulating the crush scenario of chiral auxetic structures. In the first region in which only elastic deformation occurs, close correlation can be seen; however, in the plateau region in which rotation and sliding of the chiral units, plastic deformation and failures took place besides elastic deformation, the results of FEM and experiments differ. In the plateau region, for experimental results, fluctuating behavior is more common than the FEM results. This situation can be explained by the high surface roughness and discontinuity on the surface of the chiral units which caused jamming and instantaneous lateral movement of the chiral units under crush loads, which cannot be observed in the FEM analysis. Regarding the investigation of the onset of densification, it can be said that similar behavior can be captured in the approximate points.

**Figure 18:**
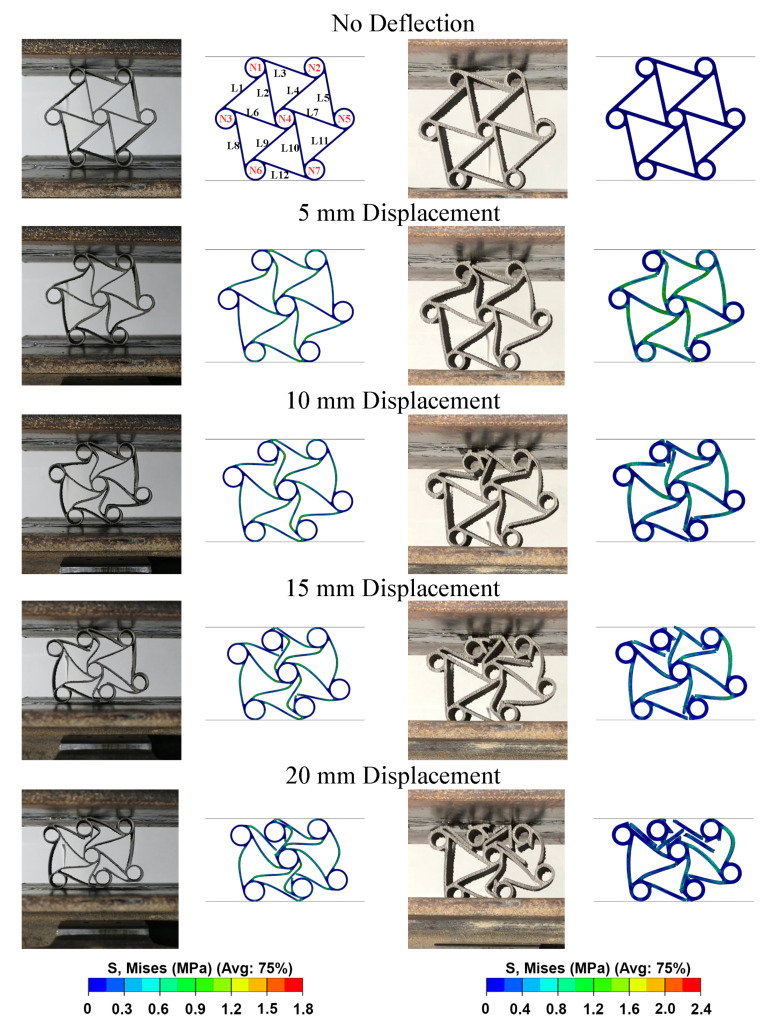
Comparison of FEM and experimental deformation stages for the crushing of chiral units with 0.8 mm and 1.6 mm thicknesses.

To have a better explanation about the compressive load–displacement curves, the deformation of chiral units had to be investigated systematically. Comparison of the experimentally and numerically deformed units can be seen with 5 mm deflection intervals in Figure 18. The ligaments and nodes were designated with letters and numbers for better explaining the deformation modes of the C1 and C2 chiral units, which can be seen in the numerical model in the no deflection stage of C1 in Figure 18. Ligaments of C1 experienced s-shape (full wave-shaped) deformation which is very useful for better energy absorption [3]; however, L8 and L5 had lower rates of bending. In C2, s-shaped deformation could not be observed due to high ligament thickness. Instead, failures occurred in the upper tips of the L1, L2 and L3; and lower tips of the L10, L11 and L12. Thus, it can be concluded that higher stress emerged in the above-mentioned points, which gives us an idea about the points and regions which must be improved for better energy absorption. Additionally, the deformation modes of the chiral units in the experiments and numerical analysis are quite similar. Under 10 mm displacement, the deformation mode is analogous with the deformation under 5 mm displacement for both C1 and C2; however, in C2, N1 becomes a separate part after losing the connection with all ligaments. Furthermore, mesh element deletion can be seen in C1, which is the sign of fracturing beginning. Under 15 mm displacement, the upper tip of the L1 and the lower tip of the L11 failed in C1. In C2, the chiral units lost their structural integrity and started to collapse. In addition, the lateral contraction of the C1 and C2 could be monitored in 5 and 10 mm deflections. However, after the failure of the ligaments started, lateral expansion or no lateral change could be seen. This also clarifies that the failures prevent auxetic behavior. Lastly, under 20 mm displacement where densification started or approached the onset of the densification point, N1, N4 and N7 localized along the same vertical line and began to touch each other. This deformation mode causes the high crush load which can be seen in Figure 17 as the densification load. As a result, by using linear elasticity and classical plasticity models in conjunction with damage initiation for ductile metals, damage evolution and element removal for ductile metal [40] specifications; and failure of the C1 and C2 auxetic chiral units under crush loads can be obtained. Plus, a close correlation can be captured.

## 5. Conclusions

The material characterization results show that Ti6Al4V components printed with EBM exhibit isotropic behavior under elastic deformation; however, anisotropic behavior is displayed under large deformations, causing plastic deformation. Regarding the effect of build orientation, the build orientation of 0${}^{\circ }$ presented with consistent and better material specifications in comparison to the components that were printed in 90${}^{\circ }$ build orientation. The strain at break values of the components that were printed in the build orientation of 0${}^{\circ }$ were reache d—4.93% strain at break value—and it was lower for a 90${}^{\circ }$ build orientation. Thus, 0${}^{\circ }$ build orientation is a preferable orientation for printing crush components. Furthermore, log signals produced during the EBM build process were investigated and we validated the stability of the production in the z direction during the process (instability would cause a non-homogenous output). Moreover, the load-carrying areas of the EBM printed parts were defined with an equation. As to the crush test, the 0.8 mm chiral element experienced a good CFE value, which is very important for whiplash issues during accidents; however, the 1.6 mm chiral element experienced a better SEA value. The reason behind this is the premature ligament failure of the 1.6 mm thickness chiral units in the plateau region caused by higher unit stiffness, and this phenomenon induced a shorter plateau region in comparison to the 0.8 mm thick chiral units. The onset of densification points occurred almost the same way for both 0.8 and 1.6 mm thick chiral units. Moreover, deformation modes were evaluated for both structures to define the high-stress regions for improvements in the topology. Failures began in nodes 1 and 7’s ligament connections for both structures unrelated to the thickness of the chiral units, which caused degradation of structural integrity.

As a result of the compressive load profile test, the resulting residual displacement at unloading can be attributed to a plastic response in the zones of the structure in which maximum stress levels occurred. Moreover, from the compressive load profile tests, the stiffness levels of two different chiral units were calculated and compared. Variations in the thickness changed the stiffness of the chiral units by the third power of the thickness ratio. Additionally, the change in ligament thickness could change the auxetic characteristics of the chiral units, and their deformation modes. The lateral deformation was not observed in the 1.6 mm thickness chiral units, so no auxeticity was observed. Furthermore, the onset of the nonlinear deformation and the onset of the yield stress were defined and validated with experimental results. According to tensile results, the material parameters of an elastic-plastic constitutive law were identified and used for the compressive load profile and crush analyses in conjunction with failure analysis. FE models were validated with experimental results. Very refined FE meshes can be required to capture all the details of the crushing, to predict damage initiation and plasticity onset in the most critical points, and the results can be very accurate.

## Figures and Tables

**Table 1:** Parameters of the chiral unit.

sinβ	cosβ	sinθ	L (mm)
0.36	0.93	0.5	26.81

**Table 2:** Chemical specifications of Arcam Ti6Al4V ELI [33].

Aluminium	Vanadium	Carbon	Iron	Oxygen	Nitrogen	Hydrogen	Titanium
6.0%	4.0%	0.03%	0.10%	0.10%	0.01%	<0.003%	Balance

**Table 3:** Corrected tensile test results for 0.8 and 1.6 mm thick tensile specimens in the orientations of 0${}^{\circ }$ and 90${}^{\circ }$.

	Young’s Modulus (GPa)	Yield Stress (MPa)	Ultimate Strength (MPa)	Strain at Failure (%)
0.8 mm 0${}^{\circ }$	110 ± 10	847 ± 5	901 ± 9	2.42 ± 0.21
0.8 mm 90${}^{\circ }$	109 ± 7	989 ± 12	1040 ± 12	1.99 ± 0.07
1.6 mm 0${}^{\circ }$	110 ± 8	855 ± 3	903 ± 1	4.93 ± 0.41
1.6 mm 90${}^{\circ }$	112 ± 8	934 ± 11	1005 ± 3	4.50 ± 0.19

**Table 4:** Dimensions of C1 and C2 by design, as-built and sandblasted.

	h (mm)	d_o_ (mm)	d_i_ (mm)	t (mm)	e (mm)
C1-Design	60.27	10.44	8.84	0.8	16
C1-As-built	60.26 ± 0.12	10.49 ± 0.12	8.30 ± 0.11	1.04 ± 0.03	16.31 ± 0.17
C1-Sandblasted	60.13 ± 0.13	10.44 ± 0.01	8.29 ± 0.13	0.97 ± 0.04	16.23 ± 0.11
C2-Design	60.27	10.44	7.24	1.6	16
C2-As-built	60.37 ± 0.07	10.49 ± 0.07	6.83 ± 0.10	1.79 ± 0.04	16.49 ± 0.05
C2-Sandblasted	60.26 ± 0.12	10.35 ± 0.04	6.82 ± 0.10	1.71 ± 0.03	16.43 ± 0.05

**Table 5:** Crush indexes for C1 and C2.

	C1				C2			
	1	2	3	4	1	2	3	4
PCF (kN)	2.65	2.64	2.53	2.67	16.01	15.73	15.53	15.20
MCF (kN)	1.51	1.28	1.35	1.69	5.21	5.40	6.17	7.01
CFE	0.57	0.48	0.53	0.63	0.32	0.34	0.39	0.46
SEA (kJ/gr)	1.14	0.94	1.01	1.31	1.94	1.97	2.46	2.35

## Data Availability

The data presented in this study are available upon request from the corresponding author.
